# Recurrent Rectal Prolapse Successfully Treated With Polyethylene Glycol

**DOI:** 10.1097/PG9.0000000000000380

**Published:** 2023-10-23

**Authors:** Regina J. Lee, Kathleen Lo, Rachel B. Schenker, Yuhua Zheng

**Affiliations:** From the * Department of Pediatrics, Children’s Hospital Los Angeles, Los Angeles, CA; † Division of Gastroenterology, Hepatology, and Nutrition, Children’s Hospital Los Angeles, Los Angeles, CA.

A previously healthy 18-month-old male presented to the emergency department for rectal prolapse. Parents endorsed 2 months of intermittent, worsening rectal bulging. History is notable for the daily consumption of 32 ounces of milk. The patient was stooling daily, eating and drinking well, and gaining weight appropriately. The successful manual reduction was administered in the ED. The patient was discharged on daily polyethylene glycol (PEG) 4.25 g daily. Prolapse improved on daily PEG. However, after PEG discontinuation, prolapse returned, significantly larger and more difficult to reduce (Figs. 1 and 2). Given the worsening prolapse, pediatric surgery performed a repeat manual reduction under sedation with flexible sigmoidoscopy. The procedure was effective, and no polyps or lead points were visualized. No biopsies were obtained during the procedure. After this manual reduction under anesthesia, the patient was adherent to a regimen of 17 g of PEG daily. No further prolapse recurrences occurred.

**Figure 1.**
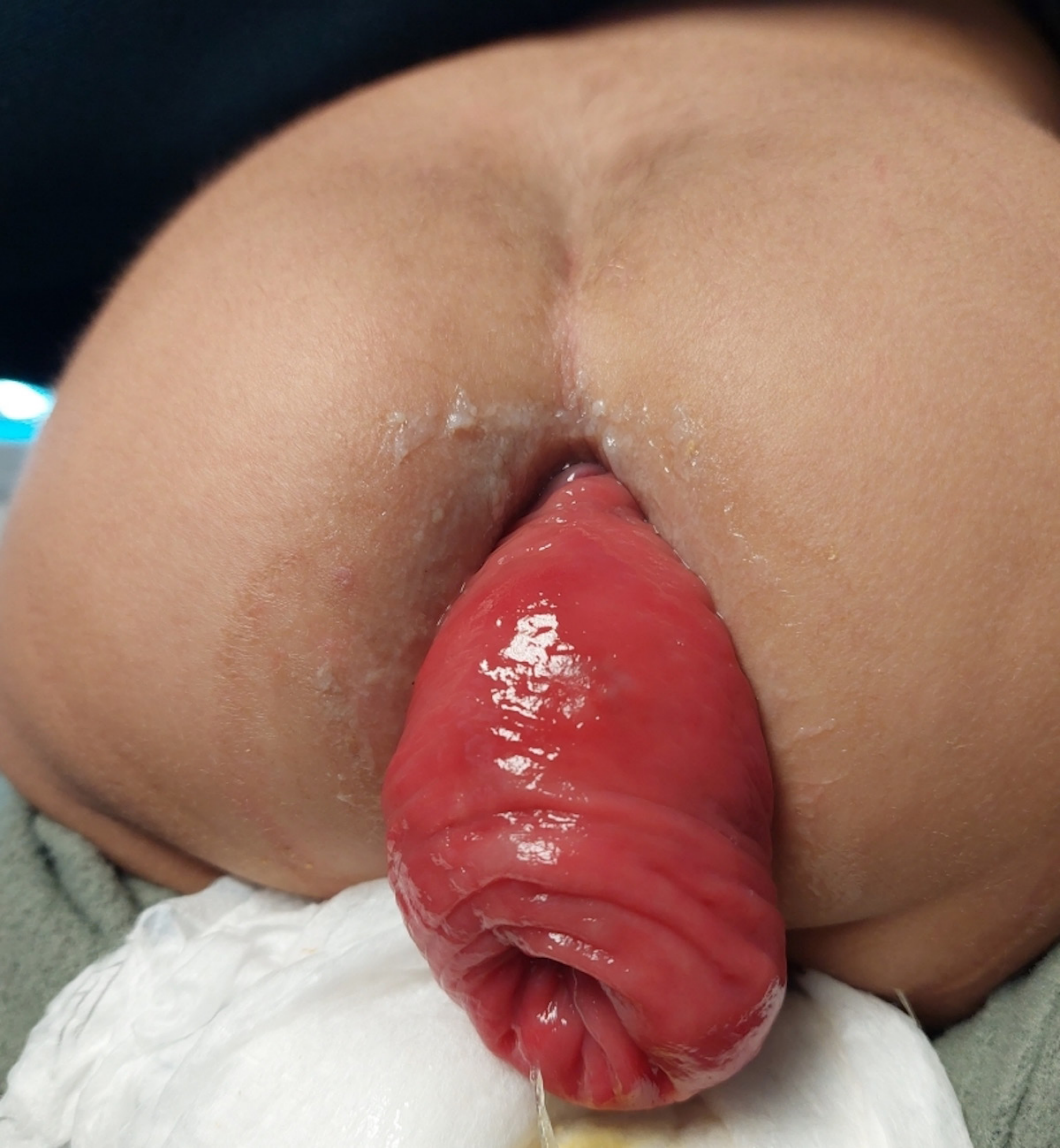
Large rectal prolapse before manual reduction.

**Figure 2.**
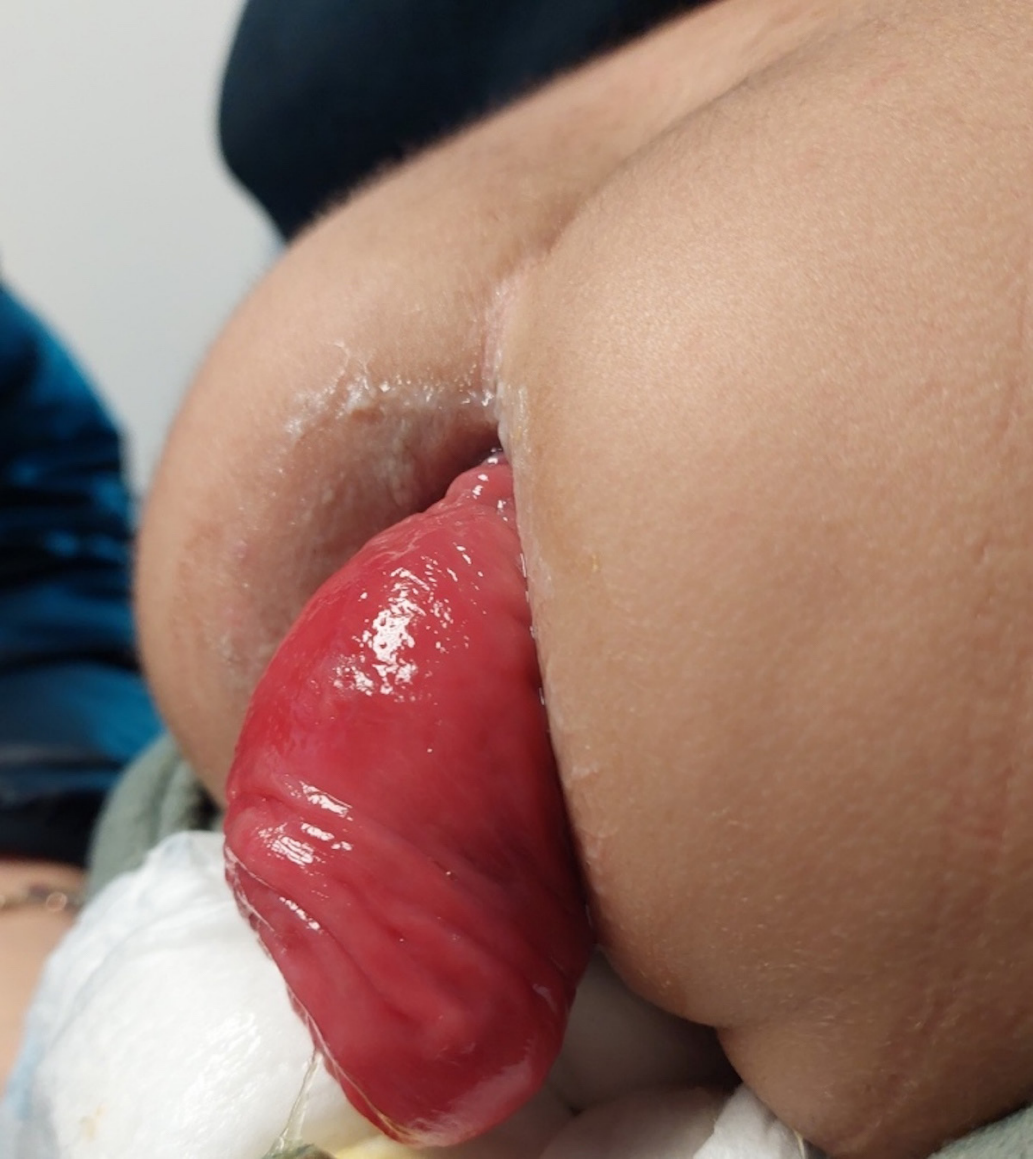
Lateral view of rectal prolapse.

Rectal prolapse is classified as partial/mucosal or complete prolapse (1). Our patient had the latter, which involves full-thickness rectal wall extrusion. Predisposing conditions include chronic constipation (most common), increased bowel motility, celiac disease, and cystic fibrosis (1–3). Additionally, there are case reports highlighting the relationship between cow’s milk protein allergy and chronic constipation, which may warrant further consideration for the reduction and/or elimination of cow’s milk in the diet (4). Since our patient’s prolapse was most likely secondary to constipation with excessive milk intake, an additional workup was not performed. In general, management for rectal prolapse involves (1) immediate manual reduction if instantaneous spontaneous reduction does not occur and (2) constipation bowel regimens. For most children, rectal prolapse resolves with a bowel regimen alone. There are no definitive indications for surgery, but it can be considered if prolapse persists despite conservative therapy or if there is difficulty in manual reduction (1–3,5).

## ACKNOWLEDGMENTS

All attempts have been exhausted in trying to contact the parents or guardian for the purpose of attaining their consent to publish the Image.
